# Analysis of Bevacizumab-Induced Gastrointestinal Perforation Using the Japanese Adverse Drug Event Report Database

**DOI:** 10.7759/cureus.78585

**Published:** 2025-02-05

**Authors:** Kana Sugishita, Mika Maezawa, Koumi Miyasaka, Sakiko Hirofuji, Moe Yamashita, Yuka Nokura, Nanaka Ichihara, Satoshi Nakao, Hideyuki Tanaka, Tomofumi Yamazaki, Kazuya Nonomura, Hirofumi Tamaki, Kazuhiro Iguchi, Mitsuhiro Nakamura

**Affiliations:** 1 Laboratory of Drug Informatics, Gifu Pharmaceutical University, Gifu, JPN; 2 Laboratory of Community Pharmacy, Gifu Pharmaceutical University, Gifu, JPN

**Keywords:** bevacizumab, gastrointestinal perforation, japanese adverse drug event report database, non-small cell lung cancer, time-to-onset analysis

## Abstract

Introduction: Bevacizumab is a recombinant humanized monoclonal antibody against human vascular endothelial growth factor (VEGF), which inhibits angiogenesis in tumor tissues by blocking VEGF activity. Although rare, bevacizumab-induced gastrointestinal perforation (BIGP) can reduce patients’ quality of life and even lead to death. We aimed to evaluate the time to BIGP onset according to the indication, its outcome, and the effect of bevacizumab in combination with various anticancer agents.

Method: Adverse events in the Japanese Adverse Drug Reaction Reports (JADER) database were defined according to the Medical Dictionary for Regulatory Activities, and “gastrointestinal perforation (SMQ ‘standardized MedDRA inquiry’ 20000107)” was extracted. Reasons for use were categorized by seven indications, and other diseases were classified as “other” and were evaluated for time-to-onset analysis and outcomes. Association rule mining was used to assess the risk of BIGP associated with the administration of bevacizumab combined with various anticancer agents.

Results: The JADER database includes 887,704 reports submitted between April 2004 and March 2024, including 2,112 reports of BIGP. The times to BIGP onset (quartile range) for non-small cell lung, colorectal, and ovarian cancers were 46.0 (11.0-122.0), 77.0 (29.0-196.0), and 67.0 (23.0-203.0) days, respectively. The log-rank test demonstrated that BIGP occurred earlier in patients with non-small cell lung cancer than in patients with colorectal (P < 0.0001) or ovarian (P = 0.0033) cancer. Association rule mining results showed that for the consequent (right-hand side) “1-100 days onset,” drugs used to treat non-small cell lung cancer were at the top. However, for “101-200 days onset,” irinotecan and drugs for colorectal cancer were at the top of the association rule. BIGP outcomes in the JADER report were 64.7% “improvement” and 35.3% “no improvement.”

Conclusion: In non-small cell lung cancer, the time to BIGP onset is earlier than that in colorectal and ovarian cancers. This finding suggests that healthcare providers can detect and intervene BIGP at early stages.

## Introduction

Bevacizumab is a recombinant humanized monoclonal antibody against human vascular endothelial growth factor (VEGF). The cytokine VEGF regulates the division and survival of vascular endothelial cells and enhances vascular permeability; its expression is elevated in cancer cells [1,2]. Bevacizumab specifically binds to human VEGF and blocks its biological activity by inhibiting its binding to VEGF receptors on vascular endothelial cells, thereby preventing tumor growth by inhibiting angiogenesis in tumor tissues [3,4]. VEGF can promote the translocation of anticancer drugs into tumor tissues by decreasing vascular permeability and interstitial pressure in these tissues [4]. Based on these pharmacological actions, bevacizumab has been approved as the world’s first angiogenesis inhibitor in the US in 2004 [5,6], and has since been used in combination with various anticancer drugs.

Adverse events (AEs) associated with bevacizumab treatment include hypertension, bleeding, and gastrointestinal perforation. Several mechanisms have been proposed for gastrointestinal perforation induced by bevacizumab. Bevacizumab inhibits VEGF, potentially causing thrombosis in smaller splanchnic or mesenteric vessels, leading to bowel ischemia and perforation [7]. VEGF supports endothelial cell proliferation, cytoprotection, and the synthesis of nitric oxide, prostacyclin, and tissue plasminogen activators while inducing clotting factors such as factor III [8]. By inhibiting VEGF, bevacizumab disrupts this balance, increasing the risk of thrombosis or bleeding. Therefore, enhanced clot formation and vasoconstriction in splanchnic vessels may result in bowel ischemia and subsequent perforation [7]. Furthermore, constant proliferation and healing of the intestinal wall depends on microcirculation, protection by nitrous oxide, prostacyclin, and normal platelet function, all of which are associated with VEGF [8]. Bevacizumab inhibits VEGF and causes gastrointestinal perforation. It may result from the combined effects of intra-abdominal inflammation and delayed wound healing associated with VEGF inhibition [6,9]. Another reason is that the intestinal wall is relatively thin at sites of ulceration, tumor necrosis, and diverticula, and inflammation in these areas can easily lead to perforation [10,11].

Bevacizumab-induced gastrointestinal perforation (BIGP), although rare, has been extensively reported, and its onset time ranges from months to years. Gastrointestinal symptoms should be noted when administering bevacizumab, regardless of the type of indication for cancer, because BIGP is a serious AE that, despite having a low probability of occurrence, can reduce a patient's quality of life and even lead to death. However, the timing of AEs based on the primary organ and their outcomes remain unclear.

Combinatorial anticancer therapies are used to enhance the efficacy of various cancer treatment regimens. Data on AE related to gastrointestinal perforation are available from original articles corresponding to individual regimens [11,12]. Polypharmacy increases the risk of AEs [13]. To the best of our knowledge, few reports have examined the effect of bevacizumab in combination with various anticancer agents on the risk of BIGP using a spontaneous reporting system (SRS).

The SRS for AEs is a collection of cases that occurred in actual clinical settings and is a useful database for pharmacovigilance. Using the SRS Japanese Adverse Drug Reaction Reports (JADER) managed by the Pharmaceuticals and Medical Devices Agency (PMDA), we evaluated the timing and outcomes of BIGP by indication and changes in the number of days of occurrence owing to the concomitant use of anticancer drugs.

## Materials and methods

Data source

The data source for AEs was the JADER database. Data were collected and fully anonymized by the PMDA. The AE reports recorded in this database were downloaded from the PMDA website [14]. The JADER data from April 2004 to March 2024 were obtained from the PMDA website. The JADER database consists of four tables: 1) DEMO (patient information, including age, gender, and reporting year), 2) DRUG (generic name, start date of administration, reason for use, and involvement in reported AE), 3) HIST (primary disease), and 4) REAC (AE, outcome, and date of AE occurrence). These four tables were integrated to create a relational database. Drugs registered in JADER are classified into three categories according to the degree of their involvement in AEs: “suspect drugs,” “concomitant drugs,” and “interactions.” In this study, only reports on “suspect drugs” were included.

Definition of AEs

AEs were coded according to the Medical Dictionary for Regulatory Activities (MedDRA), which is the terminology dictionary used in the JADER database (The International Council for Harmonization of Technical Requirements for Pharmaceuticals for Human Use “ICH”, Introductory Guide MedDRA v.23.1) [15]. To evaluate BIGP, we used a standardized MedDRA inquiry (SMQ) for gastrointestinal perforation (SMQ code: 20000107, containing 96 preferred terms) (Table 1).

**Table 1:** Ninety-six preferred terms and PT codes included in SMQ of gastrointestinal perforation*. ^*^SMQ code: 20000107. SMQ, standardized MedDRA inquiry; PT codes, Preferred Term codes.

Preferred terms	PT codes
Abdominal abscess	10060921
Abdominal hernia perforation	10074442
Abdominal wall abscess	10000099
Abscess intestinal	10000285
Acquired tracheo-oesophageal fistula	10000582
Anal abscess	10048946
Anal fistula	10002156
Anal fistula infection	10051540
Anal fistula repair	10082792
Anastomotic ulcer perforation	10002248
Anovulvar fistula	10050362
Aortoenteric fistula	10081100
Aorto-oesophageal fistula	10066870
Appendiceal abscess	10049764
Appendicitis perforated	10003012
Arterioenteric fistula	10070296
Atrio-oesophageal fistula	10075253
Chemical peritonitis	10070419
Colon fistula repair	10052931
Colonic abscess	10073573
Colonic fistula	10009995
Diverticular fistula	10013536
Diverticular perforation	10061820
Diverticulitis intestinal perforated	10084304
Douglas’ abscess	10049583
Duodenal perforation	10013832
Duodenal ulcer perforation	10013849
Duodenal ulcer perforation, obstructive	10013850
Duodenal ulcer repair	10069807
Enterocolonic fistula	10056991
Enterocutaneous fistula	10051425
Enterovesical fistula	10062570
Fistula of small intestine	10065850
Focal peritonitis	10084697
Gastric fistula	10065713
Gastric fistula repair	10071259
Gastric perforation	10017815
Gastric ulcer perforation	10017835
Gastric ulcer perforation, obstructive	10017836
Gastrointestinal anastomotic leak	10065879
Gastrointestinal fistula	10017877
Gastrointestinal fistula repair	10071258
Gastrointestinal perforation	10018001
Gastrointestinal ulcer perforation	10061975
Gastropleural fistula	10067091
Gastrosplenic fistula	10068792
Ileal perforation	10021305
Ileal ulcer perforation	10021310
Inguinal hernia perforation	10075254
Intestinal fistula	10022647
Intestinal fistula infection	10051095
Intestinal fistula repair	10052991
Intestinal perforation	10022694
Intestinal ulcer perforation	10061248
Jejunal perforation	10023174
Jejunal ulcer perforation	10023178
Large intestinal ulcer perforation	10052497
Large intestine perforation	10023804
Lower gastrointestinal perforation	10078414
Mesenteric abscess	10072408
Neonatal intestinal perforation	10074160
Oesophageal abscess	10082996
Oesophageal fistula	10065835
Oesophageal fistula repair	10058381
Oesophageal perforation	10030181
Oesophageal rupture	10052211
Oesophageal ulcer perforation	10052488
Oesophageal-pulmonary fistula	10083015
Oesophagobronchial fistula	10056992
Oesophagomediastinal fistula	10084038
Oesophagopleural fistula	10077873
Pancreatic fistula	10049192
Pancreatic fistula repair	10058384
Peptic ulcer perforation	10034354
Peptic ulcer perforation, obstructive	10034358
Perforated peptic ulcer oversewing	10034397
Perforated ulcer	10062065
Perineal abscess	10052457
Perirectal abscess	10052814
Peritoneal abscess	10034649
Peritoneocutaneous fistula	10076607
Peritonitis	10034674
Peritonitis bacterial	10062070
Pneumoperitoneum	10048299
Pneumoretroperitoneum	10068676
Procedural intestinal perforation	10074065
Rectal abscess	10048947
Rectal fistula repair	10053267
Rectal perforation	10038073
Rectoprostatic fistula	10074430
Rectourethral fistula	10066892
Retroperitoneal abscess	10038975
Small intestinal perforation	10041103
Small intestinal ulcer perforation	10052498
Umbilical hernia perforation	10066993
Upper gastrointestinal perforation	10078413

Classification of indications

Based on the information provided in the “Reasons for Use” section of the “DRUG” table in the JADER database, the indications for bevacizumab were classified into seven categories: colorectal cancer, non-small cell lung cancer, breast cancer, malignant glioma, ovarian cancer, cervical cancer, and hepatocellular carcinoma. If a disease other than them was entered, it was classified as “other” (Table 2).

**Table 2:** The number of reports for each drug use purpose. ^*^The number of purposes of drug use for each indication is as follows: colon and rectal cancer (n = 95), non-small cell lung cancer (n = 49), breast cancer (n = 16), malignant glioma (n = 10), ovarian cancer (n = 46), cervical cancer (n = 40), hepatocellular carcinoma (n = 7), and others (n = 36). ^†^The top 10 purposes of drug use by the number of reports for each indication.

Indications*	Purpose of drug use	Reports (n)^†^
Colon and rectal cancer	Colon cancer with distant metastasis	236
	Colon cancer	229
	Rectal cancer with distant metastasis	144
	Rectal cancer	111
	Recurrent rectal cancer	79
	Rectal cancer (rectal cancer)	67
	Recurrent colon cancer	55
	Colon cancer (colon cancer)	35
	Colon cancer (sigmoid colon cancer)	20
	Colon cancer (cecal cancer)	14
Non-small cell lung cancer	Lung adenocarcinoma	107
	Non-small cell lung cancer	36
	Non-small cell lung cancer (non-small cell lung cancer)	28
	Lung adenocarcinoma (lung adenocarcinoma)	13
	Non-small cell lung cancer with distant metastasis	10
	Recurrent lung adenocarcinoma	9
	Malignant neoplasms of the lung	7
	Lung adenocarcinoma, stage IV	6
	Lung adenocarcinoma (non-small cell lung cancer)	4
	Malignant neoplasm of lung (lung cancer)	4
Breast cancer	Breast cancer	25
	Breast cancer (breast cancer)	25
	Breast cancer with distant metastasis	22
	Breast cancer (right breast cancer)	5
	Recurrent breast cancer	2
	Breast cancer (left breast cancer)	2
	HER2-negative breast cancer (breast cancer)	1
	Breast cancer with distant metastasis (left breast cancer (liver, brain, bone, lymph node))	1
	Breast cancer with distant metastasis (bilateral breast cancer (multiple bone metastases, multiple liver metastases, lymph node metastases, gallbladder metastases))	1
	Recurrent breast cancer (recurrent breast cancer)	1
Malignant glioma	Malignant glioma	4
	Glioblastoma	4
	Malignant astrocytoma	2
	Glioblastoma (left thalamic glioblastoma)	2
	Malignant glioma (right frontotemporal glioblastoma)	1
	Malignant glioma (brain tumor (malignant glioblastoma))	1
	Glioblastoma (recurrent glioblastoma)	1
	Glioblastoma (glioblastoma in situ)	1
	Glioblastoma (glioblastoma)	1
	Glioma	1
Ovarian cancer	Ovarian cancer (ovarian cancer)	108
	Ovarian cancer	77
	Recurrent ovarian cancer	16
	Ovarian cancer with distant metastasis	12
	Ovarian cancer (ovarian cancer)	7
	Ovarian cancer (advanced ovarian cancer)	5
	Recurrent ovarian cancer (recurrent ovarian cancer)	3
	Ovarian cancer (advanced or recurrent ovarian cancer)	3
	Ovarian epithelial carcinoma (ovarian cancer)	2
	Ovarian cancer (ovarian cancer)	1
Cervical cancer	Cervical cancer (cervical cancer)	55
	Cervical cancer	32
	Cervical cancer (recurrent cervical cancer)	19
	Cervical cancer (cervical cancer)	10
	Cervical cancer (advanced cervical cancer)	4
	Cervical cancer (recurrent cervical cancer)	4
	Cervical cancer (advanced or recurrent cervical cancer)	3
	Cervical cancer (cervical cancer)	3
	Cervical cancer (locally advanced cervical cancer)	3
	Cervical cancer (cervical cancer)	2
Hepatocellular carcinoma	Hepatocellular carcinoma (hepatocellular carcinoma)	40
	Hepatocellular carcinoma (advanced hepatocellular carcinoma)	2
	Hepatocellular carcinoma (unresectable hepatocellular carcinoma)	2
	Hepatocellular carcinoma with distant metastasis	1
	Hepatocellular carcinoma (advanced hepatocellular carcinoma)	1
	Hepatocellular carcinoma (HCC treated)	1
	Hepatocellular carcinoma (hepatocellular carcinoma)	1
Others	Malignant neoplasms of the peritoneum (peritoneal carcinoma)	7
	Chemotherapy (chemotherapy)	6
	Peritoneal cancer with distant metastasis	4
	Gastric cancer	3
	Malignant neoplasms of the peritoneum	3
	Malignant neoplasms of unknown primary site	3
	Fallopian tube cancer (fallopian tube carcinoma)	2
	Lymph node metastasis	2
	Fallopian tube cancer	2
	Appendiceal carcinoma (appendiceal carcinoma)	2

Time-to-onset analysis

The median, quartile, and Weibull shape parameter (WSP) tests were used for time-to-onset analysis [16-18]. The WSP test is used for statistical analysis of the time-to-onset data and can describe an inconsistent ratio of AE incidence. Reports that did not include complete AE occurrence and prescription start times were excluded. The scale parameter α of the Weibull distribution determines the scale of the distribution function. A large-scale value (α) stretches the data distribution, while a small-scale value shrinks it. In the analysis of the SRSs, the shape parameter β of the Weibull distribution was used to indicate the hazard without reference populations as follows: when β was equal to 1, the hazard was estimated to be constant over time; if β was >1 and the 95% confidence interval (CI) of β excluded the value of 1, the hazard was considered to increase over time (wear-out failure type); if the upper limit of the 95% CI of β was <1, the hazard was considered to decrease over time (initial failure type). The time-to-onset profiles of BIGP were compared between stratified indication groups using the Kaplan-Meier method with the log-rank test. Statistical significance was set at P <0.05. Time-to-onset analysis was performed using JMP Pro v.17 (SAS Institute, Cary, NC).

Association rule mining

The association rule mining approach attempts to search for frequent items in databases and discovers interesting relationships between variables. Given a set of transactions T (each transaction is a set of items), an association rule can be expressed as X -> Y, where X and Y are mutually exclusive sets of items. The statistical significance and strength of the rule are measured as support and confidence. Support is defined as the percentage of transactions in the data that contain all items in both the antecedent (left-hand side: lhs) and consequent (right-hand side: rhs) of the rule. Support, confidence, and lift were the measures of statistical significance used as indicators to determine the relative strength of the rules, and these parameters were calculated as follows:

$Support=P(X \cap Y) = \frac{|X \cap Y|}{|D|}$ (1)

$Confidence=\frac {P(X\cap Y)}{P(X)}$ (2)

$Lift=\frac {P(X \cap Y)}{P(X) P(Y)}$ (3)

where D denotes the total number of transactions. Support in an itemset is defined as the proportion of transactions and shows how frequently the rule appears in the transaction. Confidence is the proportion of cases covered by the lhs of the rule that is covered by the rhs and provides an estimate of the conditional probability P (Y|X). As P (Y) appears in the denominator of the lift equation, lift can be considered as the confidence divided by P (Y). Lift can be evaluated as follows:

$lift = 1, > 1, and &lt; 1$ (4)

if X and Y are independent, positively correlated, and negatively correlated, respectively. The statistical significance of the association rule can be estimated by using the chi-squared test [19]. The chi-squared statistic is defined in terms of the support, confidence, and lift value of the single rule, which is defined by the following formula:

$Chi^2=D (lift-1)^2 \frac{(support*confidence)}{(confidence-support)(lift-confidence)}$(5)

The chi-squared statistic follows a χ^2^ distribution with 1 degree of freedom; if the significance level is set at 5%, a threshold value of the chi-squared statistic >3.84 can be used to extract valid rules. The number of days of onset of the consequent (rhs) was classified as 1-100, 101-200, 201-300, 301-400, 401-500, 501-600, 601-700, 701-800, 801-900, 901-1000, and 1001-1095 days. To efficiently extract association rules, we defined the thresholds for minimum support, confidence, and maxlen as 0.000000005, 0.000000005, and 3, respectively, based on factors such as data size and number of items. These analyses were performed using the apriori function of the arules library in the arules package of R v.4.4.1 (R Foundation for Statistical Computing, Vienna, Austria).

Outcomes

To visually evaluate the relationship between the two types of categorical data, bevacizumab-related indications (X) and outcomes (Y), a mosaic plot was constructed. Outcomes were classified as “death,” “with sequelae,” “not recovered,” “improved,” “recovered,” and “unknown.” Outcomes classified as “unknown” or blank were excluded. “No improvement” was defined as “death,” “with sequelae,” or “not recovered,” while “improvement” was defined as “improved” or “recovered.”

## Results

**Figure 1:**
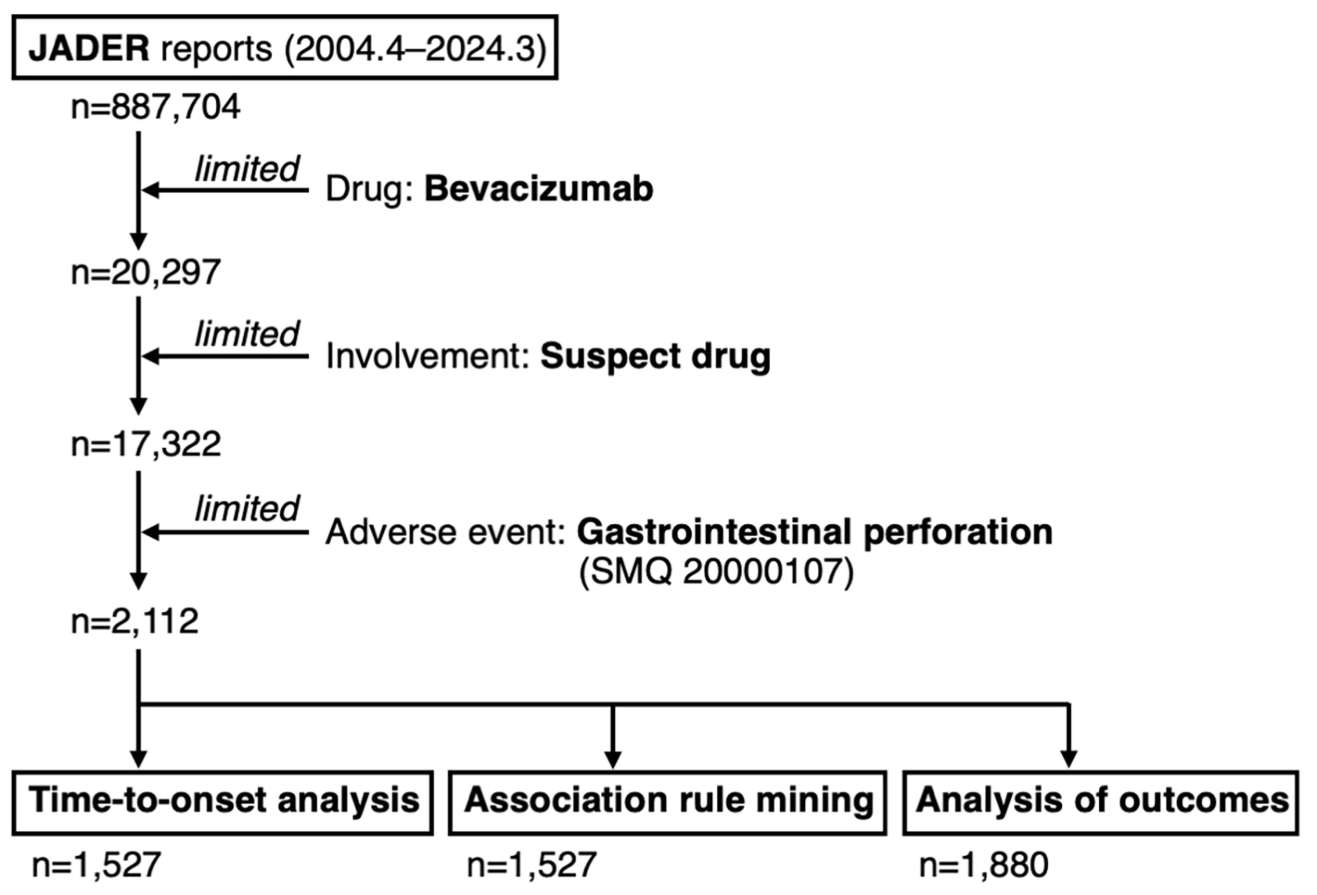
Flowchart of bevacizumab-induced gastrointestinal perforation analysis

The JADER database contains 887,704 reports submitted from April 2004 to March 2024 (Figure 1).

A total of 2,112 BIGP cases were reported, including 1,016 (49.1%) males and 1,053 (50.9%) females (Table 3). The number of reports by age <30 years, 30s, 40s, 50s, 60s, 70s, and 80s or older were 11, 34, 159, 397, 714, 463, and 86, respectively (Table 3).

**Table 3:** Reporting ratio of bevacizumab-induced gastrointestinal perforation by sex and age. ^*^Total number of reports with sex input. ^†^Total number of reports with age input.

Category	Case (n)	Reporting ratio (%)
Sex		
Total^*^	2069	100.0
Male	1016	49.1
Female	1053	50.9
Age		
Total^†^	1864	100.0
≤29 years	11	0.6
30-39 years	34	1.8
40-49 years	159	8.5
50-59 years	397	21.3
60-69 years	714	38.3
70-79 years	463	24.8
≥80 years	86	4.6

The number of reports on indications for colorectal cancer, non-small cell lung cancer, breast cancer, malignant glioma, ovarian cancer, cervical cancer, and hepatocellular cancer induced by bevacizumab administration was 1413, 329, 112, 23, 299, 182, and 52, respectively (Table 4). Gastrointestinal perforation (SMQ code: 20000107) as a percentage of all AEs for each indication of colorectal cancer, non-small cell lung cancer, breast cancer, malignant glioma, ovarian cancer, cervical cancer, and hepatocellular cancer was 12.7%, 5.1%, 5.2%, 3.1%, 16.4%, 23.7%, and 1.4%, respectively (Table 4).

**Table 4:** Reporting ratio of bevacizumab-induced gastrointestinal perforation by indication. ^*^Proportion of bevacizumab-induced gastrointestinal perforation for each indication relative to the total number (n = 2528) of bevacizumab-induced gastrointestinal perforation reports. ^†^Reporting ratio of bevacizumab-induced gastrointestinal perforation to total adverse events for each indication in patients using bevacizumab.

Indications	Total (n)	Case (n)	Proportion^*^ (%)	Reporting ratio^†^ (%)
Total		2528		
Colon and rectal cancer	11161	1413	54.2	12.7
Non-small cell lung cancer	6444	329	12.9	5.1
Breast cancer	2174	112	4.3	5.2
Malignant glioma	742	23	0.9	3.1
Ovarian cancer	1821	299	12.7	16.4
Cervical cancer	767	182	3.3	23.7
Hepatocellular carcinoma	3674	52	2.3	1.4
Others	1186	72	7.6	6.1
No entry	598	46	1.8	7.7

**Figure 2:**
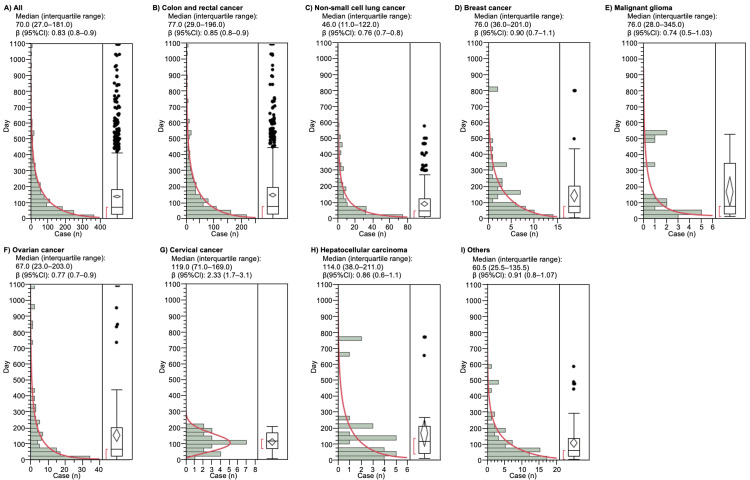
The medians and Weibull parameter of bevacizumab-induced gastrointestinal perforation. A) All, B) colon and rectal cancer, C) non-small cell lung cancer, D) breast cancer, E) malignant glioma, F) ovarian cancer, G) cervical cancer, H) hepatocellular carcinoma, and I) others. Histogram and Weibull shape parameter of chemotherapy-induced peripheral neuropathy for each drug in the ATC classification. The right panel shows box plots, which represent the median (the horizontal line within the box). The ends of the box represent the 25th and 75th quantiles, also expressed as the first and third quartile, respectively. The confidence diamond contains the mean and the upper and lower 95% CIs of the mean. The whiskers extend to the outermost data point that falls within the distances of 1.5 times the length of the inner quartiles. The bracket outside the box indicates the shortest half, which is the densest 50% of the observations. 95% CI, 95% confidence interval.

The times to BIGP onset (quartile range) for non-small cell lung, colorectal, and ovarian cancers were 46.0 (11.0-122.0), 77.0 (29.0-196.0), and 67.0 (23.0-203.0) days, respectively (Figure 2). The lower limit of the 95% CI of WSP β value for cervical cancer was >1.0, indicating a wear failure type (Table 5).

**Table 5:** The medians and Weibull parameter of each indication.

Indications	Case (n)	Median (days) (25%-75%)	Scale parameter	Shape parameter
			α (95% CI)	β (95% CI)
All	1527	70.0 (27.0-181.0)	125.8 (117.9-134.2)	0.82 (0.8-0.9)
Colon and rectal cancer	969	77.0 (29.0_196.0)	135.0 (125.6-147.2)	0.84 (0.8-0.9)
Non-small cell lung cancer	207	46.0 (11.0-122.0)	78.6 (64.7-95.0)	0.76 (0.7-0.8)
Breast cancer	67	76.0 (36.0-201.0)	138.9 (103.4-184.6)	0.90 (0.7-1.1)
Malignant glioma	19	76.0 (28.0-345.0)	136.8 (66.2-270.3)	0.73 (0.5-1.03)
Ovarian cancer	123	67.0 (23.0-203.0)	134.6 (103.9-173.1)	0.74 (0.6-0.8)
Cervical cancer	27	119.0 (71.0-169.0)	125.6 (104.3-149.8)	2.33 (1.7-3.1)
Hepatocellular carcinoma	27	114.0 (38.0-211.0)	152.9 (92.3-246.7)	0.86 (0.6-1.1)
Other	76	60.5 (25.5-135.5)	101.9 (77.5-132.7)	0.91 (0.8-1.07)

**Figure 3:**
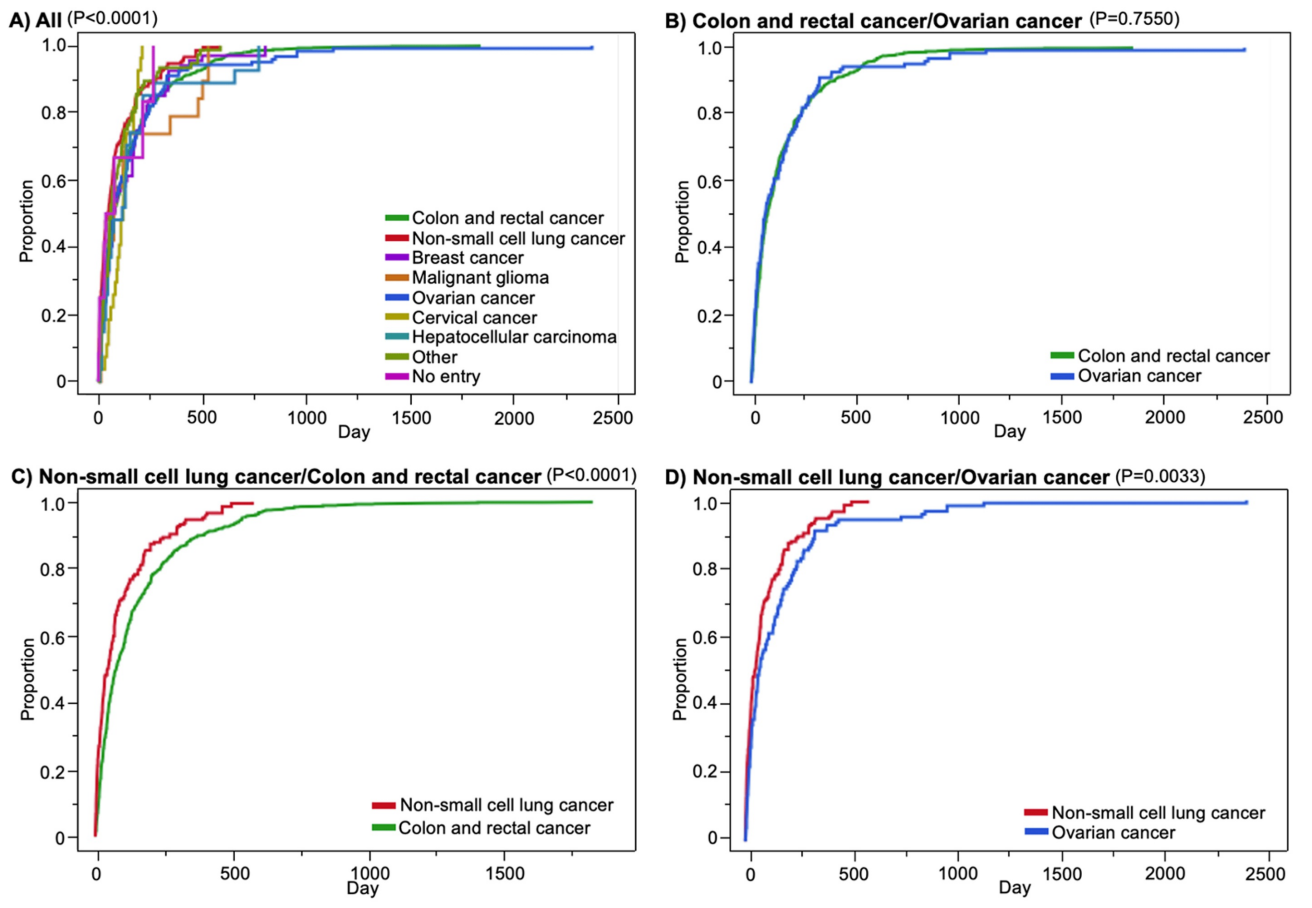
Kaplan-Meier curves of bevacizumab-induced gastrointestinal perforation for each indication. A) All indications, B) colon and rectal cancer versus ovarian cancer, C) non-small cell lung cancer versus colon and rectal cancer, and D) non-small cell lung cancer versus ovarian cancer. P-values were obtained by log-rank tests.

Kaplan-Meier curves of the time to BIGP onset for colorectal, non-small cell lung, and ovarian cancers were generated using the data of the number of days of onset for each indication (Figure 3). BIGP occurred earlier in non-small cell lung cancer than in colorectal cancer, and the log-rank test showed a significant difference in the time trends (P < 0.0001). BIGP occurred earlier in non-small cell lung cancer than in ovarian cancer, with a significant difference in the time trend according to the log-rank test (P = 0.0033).

In association rule mining, the top association rules for the consequent (rhs) of “1-100 days” included paclitaxel and carboplatin for treating non-small cell lung cancer. For the consequent (rhs) of “101-200 days,” the top associated rules included irinotecan and colorectal cancer (Table 6).

**Table 6:** Association parameters of rules†. ^*^Bevacizumab-induced gastrointestinal perforation onset. ^†^Lift value is only >1. lhs, left-hand side; rhs, right-hand side.

lhs		rhs (days*)	Support	Confidence	Coverage	Lift	Count	Chi-squared
{paclitaxel, non-small cell lung cancer}	=>	1-100	0.0362080	0.8593750	0.0421330	1.4817147	55	21.5224152
{paclitaxel, bevacizumab (genetical recombination), non-small cell lung cancer}	=>	1-100	0.0362080	0.8593750	0.0421330	1.4817147	55	21.5224152
{carboplatin, paclitaxel, non-small cell lung cancer}	=>	1-100	0.0355497	0.8571429	0.0414747	1.4778661	54	20.8346310
{carboplatin, paclitaxel, bevacizumab (genetical recombination), non-small cell lung cancer}	=>	1-100	0.0355497	0.8571429	0.0414747	1.4778661	54	20.8346310
{carboplatin, non-small cell lung cancer}	=>	1-100	0.0658328	0.7575758	0.0868993	1.3061948	100	18.8142663
{carboplatin, bevacizumab (genetical recombination), non-small cell lung cancer}	=>	1-100	0.0658328	0.7575758	0.0868993	1.3061948	100	18.8142663
{carboplatin, paclitaxel}	=>	1-100	0.0809743	0.7278107	0.1112574	1.2548744	123	17.1474559
{carboplatin, paclitaxel, bevacizumab (genetical recombination)}	=>	1-100	0.0809743	0.7278107	0.1112574	1.2548744	123	17.1474559
{non-small cell lung cancer}	=>	1-100	0.0954575	0.7073171	0.1349572	1.2195399	145	15.8554904
{bevacizumab (genetical recombination), non-small cell lung cancer}	=>	1-100	0.0954575	0.7073171	0.1349572	1.2195399	145	15.8554904
{carboplatin, ovarian cancer}	=>	1-100	0.0276498	0.7000000	0.0394997	1.2069240	42	3.71288860
{carboplatin, bevacizumab (genetical recombination), ovarian cancer}	=>	1-100	0.0276498	0.7000000	0.0394997	1.2069240	42	3.71288860
{carboplatin, paclitaxel, ovarian cancer}	=>	1-100	0.0230415	0.7000000	0.0329164	1.2069240	35	3.07301070
{carboplatin}	=>	1-100	0.1171824	0.6953125	0.1685319	1.1988419	178	16.8984425
{carboplatin, bevacizumab (genetical recombination)}	=>	1-100	0.1171824	0.6953125	0.1685319	1.1988419	178	16.8984425
{paclitaxel}	=>	1-100	0.1132324	0.6880000	0.1645820	1.1862338	172	14.4075351
{paclitaxel, bevacizumab (genetical recombination)}	=>	1-100	0.1132324	0.6880000	0.1645820	1.1862338	172	14.4075351
{paclitaxel, ovarian cancer}	=>	1-100	0.0276498	0.6774194	0.0408163	1.1679909	42	2.53219961
{paclitaxel, bevacizumab (genetical recombination), ovarian cancer}	=>	1-100	0.0276498	0.6774194	0.0408163	1.1679909	42	2.53219961
{cisplatin}	=>	1-100	0.0230415	0.6603774	0.0348914	1.1386075	35	1.46456799
{cisplatin, bevacizumab (genetical recombination)}	=>	1-100	0.0230415	0.6603774	0.0348914	1.1386075	35	1.46456799
{others}	=>	1-100	0.0329164	0.6578947	0.0500329	1.1343270	50	2.00386356
{others, bevacizumab (genetical recombination)}	=>	1-100	0.0329164	0.6578947	0.0500329	1.1343270	50	2.00386356
{capecitabine, bevacizumab (genetical recombination), colon and rectal cancer}	=>	101-200	0.0335747	0.2266667	0.1481238	1.1671412	51	1.78770118
{capecitabine, colon and rectal cancer}	=>	101-200	0.0335747	0.2256637	0.1487821	1.1619769	51	1.68770180
{capecitabine, bevacizumab (genetical recombination)}	=>	101-200	0.0335747	0.2188841	0.1533904	1.1270677	51	1.07662617
{capecitabine}	=>	101-200	0.0335747	0.2179487	0.1540487	1.1222512	51	1.00160993
{bevacizumab (genetical recombination), colon and rectal cancer}	=>	101-200	0.1263990	0.2012579	0.6280448	1.0363074	192	0.81916222
{colorectal cancer}	=>	101-200	0.1263990	0.1991701	0.6346280	1.0255574	192	0.41753900
{oxaliplatin, bevacizumab (genetical recombination), colon and rectal cancer}	=>	101-200	0.0901909	0.1974063	0.4568795	1.0164754	137	0.08403440
{bevacizumab (genetical recombination)}	=>	101-200	0.1942067	0.1957532	0.9921001	1.0079628	295	2.93050471
{oxaliplatin, colon and rectal cancer}	=>	101-200	0.0901909	0.1957143	0.4608295	1.0077627	137	0.01895481
{oxaliplatin, bevacizumab (genetical recombination)}	=>	101-200	0.0908492	0.1946403	0.4667544	1.0022328	138	0.00160598
{irinotecan hydrochloride hydrate, fluorouracil, bevacizumab (genetical recombination), colon and rectal cancer}	=>	201-300	0.0322581	0.1877395	0.1718236	1.9667327	49	31.2458918
{irinotecan hydrochloride hydrate, fluorouracil, colon and rectal cancer}	=>	201-300	0.0322581	0.1863118	0.1731402	1.9517766	49	30.5672510
{irinotecan hydrochloride hydrate, fluorouracil, bevacizumab (genetical recombination)}	=>	201-300	0.0322581	0.1863118	0.1731402	1.9517766	49	30.5672510
{irinotecan hydrochloride hydrate, fluorouracil}	=>	201-300	0.0322581	0.1849057	0.1744569	1.9370462	49	29.9013349
{irinotecan hydrochloride hydrate, bevacizumab (genetical recombination), calcium levofolinate, colon and rectal cancer}	=>	201-300	0.0296248	0.1785714	0.1658986	1.8706897	45	24.2980253
{irinotecan hydrochloride hydrate, fluorouracil, bevacizumab (genetical recombination), calcium levofolinate, colon and rectal cancer}	=>	201-300	0.0296248	0.1785714	0.1658986	1.8706897	45	24.2980253
{irinotecan hydrochloride hydrate, calcium levofolinate, colon and rectal cancer}	=>	201-300	0.0296248	0.1778656	0.1665570	1.8632956	45	24.0008227
{irinotecan hydrochloride hydrate, fluorouracil, calcium levofolinate, colon and rectal cancer}	=>	201-300	0.0296248	0.1778656	0.1665570	1.8632956	45	24.0008227
{irinotecan hydrochloride hydrate, bevacizumab (genetical recombination), calcium levofolinate}	=>	201-300	0.0296248	0.1771654	0.1672153	1.8559598	45	23.7066498
{irinotecan hydrochloride hydrate, fluorouracil, bevacizumab (genetical recombination), calcium levofolinate}	=>	201-300	0.0296248	0.1771654	0.1672153	1.8559598	45	23.7066498
{irinotecan hydrochloride hydrate, calcium levofolinate}	=>	201-300	0.0296248	0.1764706	0.1678736	1.8486815	45	23.4154678
{irinotecan hydrochloride hydrate, fluorouracil, calcium levofolinate}	=>	201-300	0.0296248	0.1764706	0.1678736	1.8486815	45	23.4154678
{irinotecan hydrochloride hydrate, bevacizumab (genetical recombination), colon and rectal cancer}	=>	201-300	0.0355497	0.1719745	0.2067149	1.8015814	54	26.9810040
{irinotecan hydrochloride hydrate, colon and rectal cancer}	=>	201-300	0.0355497	0.1703470	0.2086899	1.7845317	54	26.1574883
{irinotecan hydrochloride hydrate, bevacizumab (genetical recombination)}	=>	201-300	0.0355497	0.1677019	0.2119816	1.7568216	54	24.8295589
{irinotecan hydrochloride hydrate}	=>	201-300	0.0355497	0.1661539	0.2139566	1.7406048	54	24.0587090
{fluorouracil, bevacizumab (genetical recombination), colon and rectal cancer}	=>	201-300	0.0487163	0.1134969	0.4292298	1.1889782	74	4.32785020
{fluorouracil, bevacizumab (genetical recombination)}	=>	201-300	0.0493746	0.1134645	0.4351547	1.1886379	75	4.41766117
{fluorouracil, colon and rectal cancer}	=>	201-300	0.0487163	0.1124620	0.4331797	1.1781365	74	3.90794860
{fluorouracil}	=>	201-300	0.0493746	0.1124438	0.4391047	1.1779455	75	3.99466705
{fluorouracil, bevacizumab (genetical recombination), calcium levofolinate}	=>	201-300	0.0467413	0.1100775	0.4246215	1.1531569	71	2.78959896
{fluorouracil, bevacizumab (genetical recombination), calcium levofolinate, colon and rectal cancer}	=>	201-300	0.0460830	0.1100629	0.4186965	1.1530037	70	2.71719370
{bevacizumab (genetical recombination), calcium levofolinate}	=>	201-300	0.0467413	0.1099071	0.4252798	1.1513718	71	2.73230134
{bevacizumab (genetical recombination), calcium levofolinate, colon and rectal cancer}	=>	201-300	0.0460830	0.1098901	0.4193548	1.1511936	70	2.66046773
{fluorouracil, calcium levofolinate}	=>	201-300	0.0467413	0.1093991	0.4272548	1.1460496	71	2.56416838
{fluorouracil, calcium levofolinate, colon and rectal cancer}	=>	201-300	0.0460830	0.1093750	0.4213298	1.1457974	70	2.49408345
{calcium levofolinate}	=>	201-300	0.0467413	0.1092308	0.4279131	1.1442865	71	2.50937355
{calcium levofolinate, colon and rectal cancer}	=>	201-300	0.0460830	0.1092044	0.4219882	1.1440099	70	2.43988032
{bevacizumab (genetical recombination), colon and rectal cancer}	=>	201-300	0.0638578	0.1016772	0.6280448	1.0651558	97	1.15511803
{oxaliplatin, fluorouracil, bevacizumab (genetical recombination)}	=>	201-300	0.0335747	0.1013917	0.3311389	1.0621649	51	0.30830820
{oxaliplatin, fluorouracil, bevacizumab (genetical recombination), colon and rectal cancer}	=>	201-300	0.0329164	0.1010101	0.3258723	1.0581679	50	0.26356775
{colorectal cancer}	=>	201-300	0.0638578	0.1006224	0.6346280	1.0541065	97	0.81941361
{oxaliplatin, fluorouracil}	=>	201-300	0.0335747	0.1003937	0.3344306	1.0517106	51	0.21651700
{oxaliplatin, bevacizumab (genetical recombination)}	=>	201-300	0.0467413	0.1001410	0.4667544	1.0490638	71	0.33955015
{oxaliplatin, fluorouracil, colon and rectal cancer}	=>	201-300	0.0329164	0.1000000	0.3291639	1.0475862	50	0.17905157
{oxaliplatin, bevacizumab (genetical recombination), colon and rectal cancer}	=>	201-300	0.0454246	0.0994236	0.4568795	1.0415482	69	0.23400776
{oxaliplatin}	=>	201-300	0.0467413	0.0993007	0.4707044	1.0402604	71	0.23228787
{oxaliplatin, colon and rectal cancer}	=>	201-300	0.0454246	0.0985714	0.4608295	1.0326207	69	0.14656186
{bevacizumab (genetical recombination)}	=>	201-300	0.0954575	0.0962177	0.9921001	1.0079628	145	1.28317452
{oxaliplatin, bevacizumab (genetical recombination), calcium levofolinate}	=>	201-300	0.0309414	0.0959184	0.3225807	1.0048276	47	0.00178840
{oxaliplatin, fluorouracil, bevacizumab (genetical recombination), calcium levofolinate}	=>	201-300	0.0309414	0.0959184	0.3225807	1.0048276	47	0.00178840

**Figure 4:**
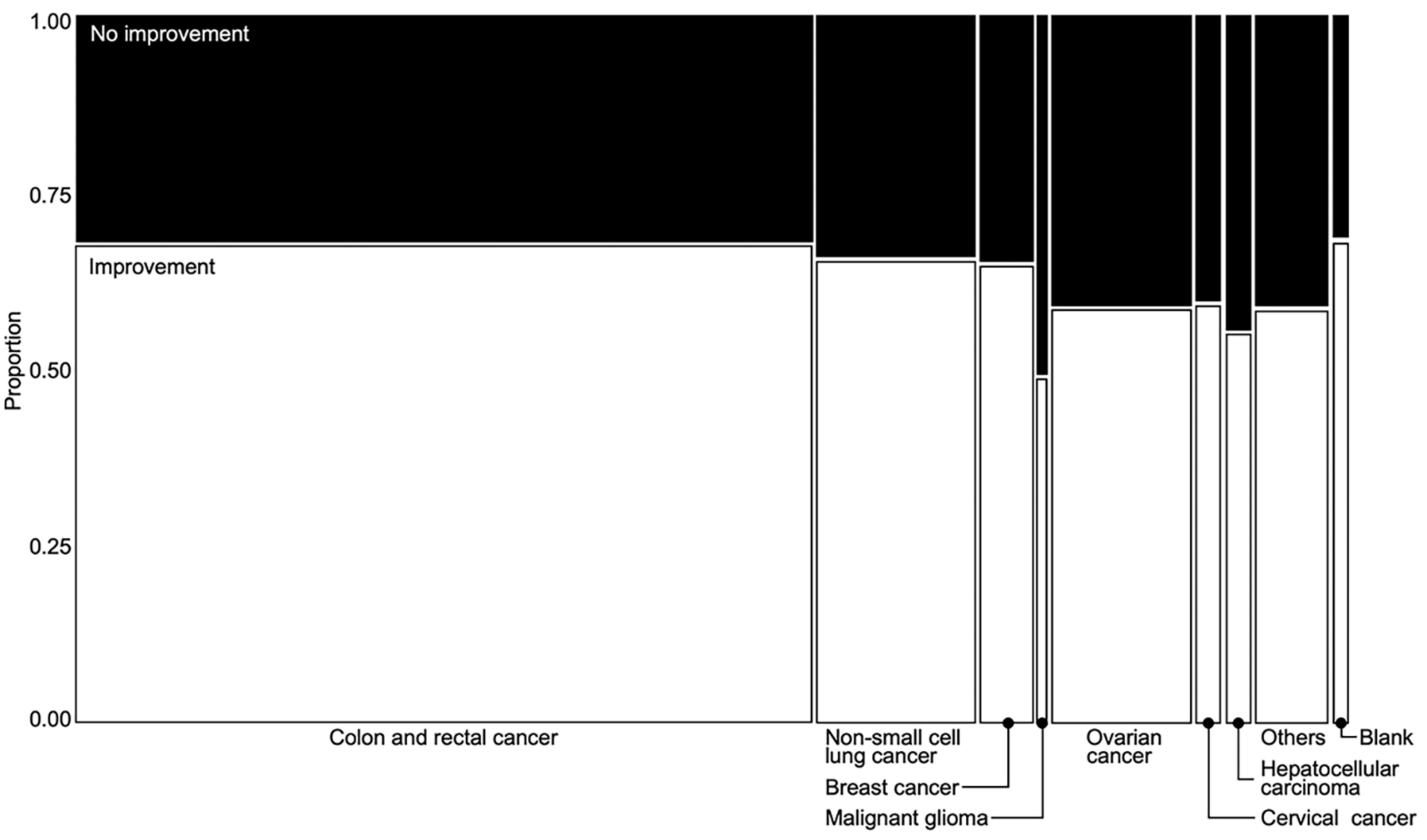
Mosaic plot of outcomes of bevacizumab-induced gastrointestinal perforation. The width of the mosaic diagram is proportional to the sum of the frequencies of each stratum of the category, which is the proportion of factors present or absent. The height of the rectangle in the bar is proportional to the frequency of each stratum of the category, which is the proportion of “no improvement” to “improvement.” The black rectangle represents “no improvement” and the white rectangle represents “improvement.”

In terms of the outcome of BIGP in the reports of the JADER database, 64.7% of the respondents mentioned an improvement, whereas 35.3% mentioned no improvement. The seven indications and BIGP outcomes are summarized in Figure 4. “No improvement” was noticed in >50% of cases in malignant gliomas and >30% of cases in the other categories.

## Discussion

BIGP is a rare but serious adverse effect, which can lead to death. The most common age of onset of BIGP is during the 60s [12], which is consistent with the results of the present study. Our results showed that cervical cancer had the highest incidence among primary cancers associated with BIGP. Radiotherapy is the standard treatment for cervical cancer, and radiation therapy is a risk factor for BIGP [10,20]. This result may have been partially influenced by the combination of radiation therapy and bevacizumab. However, as the JADER database does not contain information on the presence or absence of radiotherapy, we were unable to examine this further.

In previous studies, BIGP in colorectal cancer occurred within six months of initiation of bevacizumab administration (median, 100.5 days) [21]. In the present study, most BIGPs in colorectal cancer occurred within 196 days of starting bevacizumab treatment (median, 77 days).

The time to BIGP onset in patients with non-small cell lung cancer was significantly shorter than that in patients with colorectal and ovarian cancers. Association rule mining results also demonstrated relatively high non-small cell lung cancer-related terms in lhs when rhs was 1-100 days, suggesting that non-small cell lung cancer was associated with a relatively low number of days of BIGP. Paclitaxel, carboplatin, erlotinib, and atezolizumab are used in combination with bevacizumab in regimens related to non-small cell lung cancer and are known to carry the risk of gastrointestinal perforation alone [22-29]. Therefore, BIGP may have developed early because these malignant agents were combined with bevacizumab. Furthermore, the dose of bevacizumab in the non-small cell lung cancer regimen is 15 mg/kg, compared to 5 mg/kg for colorectal cancer and 10 mg/kg for ovarian cancer. The earlier onset of BIGP may partly result from these higher doses compared to other cancer indications [30,31].

Gastrointestinal perforation typically manifests suddenly and dramatically, presenting as acute abdomen with severe generalized abdominal pain, tenderness, and peritoneal signs [32]. The pain may occasionally radiate to the shoulder [32]. In patients with non-small cell lung cancer and other conditions, early recognition of these clinical symptoms may facilitate the timely identification and intervention of BIGP.

Drugs known to cause BIGP include barium sulfate containing X-ray contrast media; diazepines, oxazepines, thiazepines, and oxepines; drugs for treating hyperkalemia and hyperphosphatemia; nonselective monoamine reuptake inhibitors, and oral bowel cleansers [33]. In the present study, these drugs were rarely used in combination for treating non-small cell lung cancer (data not shown).

In association rule mining, among the anticancer agents included in the antecedent (lhs), those with gastrointestinal perforation (SMQ 20000107) listed in the package insert were paclitaxel (<0.1%), carboplatin (frequency unknown), and cisplatin (frequency unknown) [22-25,34,35]. Intestinal perforation (PT10022694) was caused by irinotecan (unknown frequency) [36,37]. The listed AEs included gastrointestinal ulcer (SMQ 20000106) which is considered a risk factor for gastrointestinal perforation, fluorouracil (frequency unknown), and calcium levofolinate (<0.1-5%) [38-41]. As drugs with AEs listed in Perforation of the Gastrointestinal Tract (SMQ 20000107) appear in the antecedent (lhs), the risk of developing BIGP may increase by the concomitant use of these drugs with bevacizumab.

In terms of the outcome by indication, “no improvement” exceeded 50% for malignant gliomas. This may be due to the fact that malignant gliomas have a worse prognosis than other indications [42]. However, it should be noted that the percentage of reported outcomes is the percentage of spontaneous reports of AEs, and it does not indicate a true prognosis.

The risk factors for BIGP include concomitant inflammation of the gastrointestinal tract or other abdominal cavities, radiation therapy, and prior chemotherapy with ≥3 regimens in ovarian cancer [30,43]. In general, bevacizumab should not be readministered to patients with a history of gastrointestinal perforation to prevent the recurrence of this severe complication [10]. As AEs of administering anticancer agents are influenced by the type of cancer, treatment modality, and regimen, evaluating each risk factor is important. However, the SRS lacks detailed information on patient background and drug administration; therefore, inferring regimens is impossible. Consequently, regimen-related analyses were not performed in this study.

Limitations

The JADER database is a voluntary reporting system and is not suitable for accurate risk assessment owing to overreporting, underreporting, missing data, lack of detailed information on patient background, and effects of confounding factors and biases. Most notably, there is a lack of comparison groups. As an SRS does not indicate the risk of AE occurrence in absolute terms and can only offer a rough indication of signal strength, it should be interpreted with caution. Furthermore, it is known that in time-to-onset studies with long observation periods, various unknown factors are likely to influence the occurrence of the subject event [16]. The incidence of BIGP after prescription depends on the causal mechanism and often varies over time. As the observation period of this study was more than three years, attention should be paid to the presence of factors other than bevacizumab that affect BIGP. Although epidemiological studies may be needed for confirmation, our results, based on JADER’s assessment, are consistent with previous reports and are believed to provide practical information to better understand this issue. The findings may be useful in developing protocols for future clinical trials and guidelines for drug administration.

## Conclusions

Information on the time to BIGP onset, outcomes, and risks associated with various concomitant anticancer drugs analyzed using the SRS data can help healthcare providers in the early identification of and intervention in BIGP.
